# Point-of-care testing in India: missed opportunities to realize the true potential of point-of-care testing programs

**DOI:** 10.1186/s12913-015-1223-3

**Published:** 2015-12-14

**Authors:** Nora Engel, Gayatri Ganesh, Mamata Patil, Vijayashree Yellappa, Caroline Vadnais, Nitika Pant Pai, Madhukar Pai

**Affiliations:** Department of Health, Ethics & Society, Research School for Public Health and Primary Care, Maastricht University, Postbus 616, Maastricht, MD NL - 6200 The Netherlands; Institute of Public Health, #250, 2nd C Main, 2nd C Cross, Girinagar I Phase, Bangalore, 560 085 India; Department of Epidemiology & Biostatistics, McGill International TB Centre, McGill University, 1020 Pine Ave West, Montreal, QC H3A 1A2 Canada; Division of Clinical Epidemiology, Department of Medicine, McGill University and McGill University Health Centre, V Building, Royal Victoria Hospital, 687 Pine Avenue West, Montreal, H3A1A1 Canada

**Keywords:** Point-of-care, Testing, Diagnostics, India, Qualitative

## Abstract

**Background:**

The core objective of any point-of-care (POC) testing program is to ensure that testing will result in an actionable management decision (e.g. referral, confirmatory test, treatment), within the same clinical encounter (e.g. POC continuum). This can but does not have to involve rapid tests. Most studies on POC testing focus on one specific test and disease in a particular healthcare setting. This paper describes the actors, technologies and practices involved in diagnosing major diseases in five Indian settings – the home, community, clinics, peripheral laboratories and hospitals. The aim was to understand how tests are used and fit into the health system and with what implications for the POC continuum.

**Methods:**

The paper reports on a qualitative study including 78 semi-structured interviews and 13 focus group discussions with doctors, nurses, patients, lab technicians, program officers and informal providers, conducted between January and June 2013 in rural and urban Karnataka, South India. Actors, diseases, tests and diagnostic processes were mapped for each of the five settings and analyzed with regard to whether and how POC continuums are being ensured.

**Results:**

Successful POC testing hardly occurs in any of the five settings. In hospitals and public clinics, most of the rapid tests are used in laboratories where either the single patient encounter advantage is not realized or the rapidity is compromised. Lab-based testing in a context of manpower and equipment shortages leads to delays. In smaller peripheral laboratories and private clinics with shorter turn-around-times, rapid tests are unavailable or too costly. Here providers find alternative measures to ensure the POC continuum. In the home setting, patients who can afford a test are not/do not feel empowered to use those devices.

**Conclusion:**

These results show that there is much diagnostic delay that deters the POC continuum. Existing rapid tests are currently not translated into treatment decisions rapidly or are not available where they could ensure shorter turn-around times, thus undermining their full potential. To ensure the success of POC testing programs, test developers, decision-makers and funders need to account for such ground realities and overcome barriers to POC testing programs.

**Electronic supplementary material:**

The online version of this article (doi:10.1186/s12913-015-1223-3) contains supplementary material, which is available to authorized users.

## Background

Point-of-care (POC) tests have been hailed as an efficient and innovative way to manage infectious diseases, particularly in resource-poor settings, mainly to shorten diagnostic delay and treatment initiation. Despite the many definitions of POC testing [1–5], they all include the critical elements of rapid turn-around-times (TATs) to allow for quick diagnosis, and referral or treatment decisions completed within the same patient encounter (i.e. POC continuum) or at the very minimum, with results delivered on the same day. In a recent article, we proposed a framework that envisions POC testing as programs, rather than just tests, across five settings (home, community, peripheral laboratory, clinic, and hospital) [1]. POC testing understood in this way can include a spectrum of technologies (simplest to more sophisticated), users (lay persons to highly trained), and settings. What matters is how tests are implemented in the health system. This framework recognizes the inter-dependence of different parts of the health system [6] and the need for dialogue and policy consonance between health systems development and disease control strategies [7].

Most articles on POC testing focus on cost-effectiveness, feasibility, and acceptability related to one specific disease in one of these settings [2–5, 8]. Recent exceptions are studies on combined testing strategies for sexually transmitted infections including HIV and syphilis [9, 10]. However, diseases do not occur in isolation, diagnostic tests are not always conducted separately and settings are often interlinked. Patients present with multiple or unspecific symptoms, at different levels of care (in clinics, health posts, laboratories or hospitals), in need of several diagnostic tests in parallel that require interaction among different types of healthcare workers. Therefore, testing needs to fit into a variety of existing daily work flow and care processes, and calls have been made for more operational research into health system requirements and impact of technologies on diagnostic cycles and delays [11–13].

In order to develop new POC tests that function in such dynamic and complex health systems, we need to understand how major diseases are diagnosed and technologies for testing are used at different points of care and to what effect [14]. In order to address this research gap, we have studied how POC testing occurs across five different settings in India and for a variety of major, largely infectious, diseases. We examined the types of prevalent diseases, what is being diagnosed, by whom and when, the TATs involved and how test and treat cycles are being completed. The aim was to comprehensively describe how tests are used, by whom and how they fit into the Indian health system and with what implications for the POC continuum.

## Methods

Data were collected as part of a qualitative research project on barriers to POC testing. Data collection took place between January and June 2013 in Bangalore, an urban setting, and Tumkur, a rural district in Karnataka (India). The urban setting is a predominantly poor neighborhood, including one area that is considered a slum, with a population of around 44,500 individuals spread over 2.7 square km. Available healthcare services in the area include two government health centres providing outpatient care and outreach services, and 32 private providers from various systems of medicine including Unani, Ayurveda, allopathy and homeopathy. The rural setting is located 70 km outside Bangalore with an estimated population of 2.7millions spread over 10,600 square km of which 80 % live in villages. The area includes a public district hospital, nine sub-district hospitals, 160 primary health centres (PHCs) and a range of private providers from informal to highly specialized ones.

### Data collection

Seventy eight semi-structured interviews were conducted with healthcare providers (doctors, nurses, specialists, traditional healers and informal providers), patients, community health workers (CHWs), test manufacturers, laboratory technicians, managers and policy-makers. Participants were purposively sampled to represent the five settings of hospitals, peripheral laboratories, clinics, communities and homes in both the public/private sector and rural/urban settings (Table 1). Additionally, 13 focus group discussions (FGDs) were conducted with tuberculosis (TB) and diabetic patients, CHWs, laboratory technicians, TB program staff and medical officers at primary care clinics, and with hospital nurses, all of which were selected on a convenience basis to represent the different settings. The total number of FGD participants was 94, with a median group size of six.

**Table 1:** Participant overview per setting

Setting	Type of participant	No. of interviewed participants (interview code)				Total interviews	No. of FGDs (FGD code)
		Urban public	Urban private	Rural public	Rural private		
Home	Tuberculosis (TB); Diabetes Mellitus (DM); Typhoid (TP) patients	2 (TB patient #2, 4)	2 (TB patient #1, DM patient #1)	1 (TB patient #3)	2 (TB & DM patient #5, TP patient #1)	7	3 (FGD #4, 10 - DM patients, FGD #5 - TB patients)
Community	Community Health Worker (CHW); Auxiliary-Nurse Midwife (ANM); Accredited Social Health Activist (ASHA); Link Worker (LW); Community Health Assistant (CHA)	0	0	2 (CHW #1, 2)	0	2	5 (FGD #2 – ANM, FGD #3 – ASHA, FGD #7 – LW, FGD #8 – ANM, FGD #13 - CHA)
Clinic	Specialist doctor (SP); Hospital Manager (HM); Private practitioner (PP); Medical Officer (MO) Ayush Practitoner (AP)	2 (SP #6, HM #2)	6 (PP #2, 3, 4, 11, 13, AP #1)	2 (MO #1, 2)	9 (PP #1, 6, 7, 8, 9, 10, 12, 14, 15)	19	1 (FGD #6 - MO)
Peripheral lab (stand-alone)	Lab technician (LT); Lab Material Distributer (LMD);		4 (LT #6, 7, 8, LMD #1)		10 (LT #3, 4, 10, 11, 12, 13, 14, 15, 17, 18,)	14	1 (FGD #9 -LT)
Peripheral lab (attached to clinic)	Lab technician (LT); Lab Manager (LM); Lab Specialist (LSP)	1 (LT #5)	1 (LM #2)	3 (LT #2, 19, 20)	3 (LT #1, LSP #4, 5)	8	
Hospital	Specialist provider (SP)	1 (SP #14)	5 (SP #8, 10, 11, 12, 13)	4 (SP #1, 2, 3, 15)	3 (SP #7, 9, 16)	13	0
	Hospital Manager (HM); Program Officer (PO); Private practitioner (PP)	0	1 (PP #5)	5 (PO #1, 2, 3, 4; HM #1)	0	6	2 (FGD #11, 12 - TB PO)
	Staff Nurse (SN)	0	0	5 (SN #1, 2, 3, 4, 5)	0	5	1 (FGD #1 -SN)
	Lab technician (LT); Lab Manager (LM)	0	3 (LT 9#, LM #1, 3)	1 (LT #16)	0	4	0
Total						78	13

Legend Table 1:

Home: *TB* tuberculosis, *DM* diabetes mellitus, *TP* typhoid patients

Community: *ANM* auxiliary-nurse midwife, *ASHA* accredited social health activist, *CHA* community health assistant, *CHW* community health worker, *LW* link worker

Clinic: *AP* ayush practitioner, *HM* hospital manager, *MO* medical officer, *PP* private practitioner, *SP* specialist doctor

Peripheral lab: *LM* lab manager, *LMD* lab material distributer, *LT* lab technician, *LSP* lab specialist

Hospital: *HM* hospital manager, *LT* lab technician, *LM* lab manager, *PP* private practitioner, *PO* program officer, *SN* staff nurse, *SP* specialist provider

The semi-structured interviews and FGDs were conducted jointly by Patil (MP) (a public health scientist and physician) and Engel (NE) (a social scientist). The topics explored included diagnostic processes for the major diseases per setting (mainly HIV, TB, malaria, hepatitis, syphilis, diabetes, typhoid and dengue) and challenges therein, understanding of diagnosis, and visions of an ideal test. The interviews specifically examined diagnostic steps for each major disease occurring in the setting in great detail from ordering a test to acting on a result, including available material and capacities, TATs, and referral processes (Additional file 1). The FGDs focused exclusively on challenges experienced when diagnosing. Interview and FGD guides were piloted and revised during the fieldwork to improve clarity of questions and translated into a local language (Kannada). Interviews and discussions were held in either English or Kannada, depending on the preference of the participants. Aside from seven interviewees who refused, all interviews and discussions were digitally recorded, and the note taker wrote down main points raised, non-verbal communication and setting characteristics.

### Data entry and analysis

Audio files and notes were transcribed and, if applicable, translated into English. MP then checked and edited those files, which were then cross-checked by NE. Data analysis was done using Nvivo 9 (QSR International). The study team jointly devised a coding scheme and coded the material in close communication with each other, further grouping material into emerging topics and themes in an iterative manner [15–17]. For this paper and in line with the above framework, actors, diseases, tests and diagnostic processes were mapped for each of the five settings and analyzed with regard to whether and how POC continuums are being ensured. Below, we have ordered our material along these five settings and have discussed the cross-cutting themes of diagnostic processes and implications for the POC continuum in each of these settings. A detailed analysis of the themes that emerged on challenges in diagnosing at the POC is published elsewhere [18]. In this paper, professional roles are used to mask study participants’ identity.

Like every qualitative interview and group discussion based study, we rely on statements by interviewees which might have been biased (e.g. social desirability). We enhanced rigor by triangulating findings across different informants and methods of data collection (e.g. observations, interviews, FGDs). Furthermore, the joint data collection and analysis of the collected material allowed combining different professional perspectives.

### Ethics

The study was approved by the ethics review board of the Institute of Public Health, Bangalore, India, and the ethics review board of the McGill University Health Centre (MUHC), Montreal, Canada. Approvals for interviews and discussions were sought from district and local authorities as necessary. All participants were provided with information sheets (in either English or Kannada) explaining the objectives of the study and all but the participants of FGD #10 diabetic patients (who agreed to participation verbally) signed informed consent forms prior to participation.

## Results and discussion

The results (see Table 2 for an overview) describe how diagnostic tests are used, by whom and with what implications for ensuring the POC continuum in five settings: the home, the community, public and private sector clinics, peripheral laboratories and hospitals. Each setting employs different technologies and involves different actors that influence diagnostic practices, and therefore results are presented and discussed for their implications for each setting separately.

**Table 2:** Testing in home, community, clinic, peripheral lab and hospital settings

Setting	Tests done	By whom and where	Completion of POC test and treat cycle	Implications for POC continuum
Home	Glucometer, urine pregnancy	Affluent, educated only, done in home or tests bought and taken to doctor	Confirm urine pregnancy test with doctor/lab; visit local doctor/lab directly for monthly monitoring or if have symptoms of high/low sugar;	Patients not able/empowered to use diagnostic devices
Community	Malaria smear (or rapid test if endemic); urine pregnancy	ASHA, at doorstep	Patient and sample referred to PHC; results communicated to CHW (over phone), CHWs visit patient to encourage to seek treatment if transport/ workload permit	Diagnostic devices cannot overcome health system challenges (equipment, infrastructure) that undermine testing at doorstep
	Glucometer readings (as part of pilot program)	ANM, at camp, anganwadi center or doorstep		
	Haemoglobin using Haemoglobinometer.	ASHA & ANM, same as above		
	Sputum and malaria smear sample	ASHA &ANM, same as above		
	HIV rapid and HIV Coombs’	HIV mobile testing van		
Clinic (public sector)	Malaria smear; HbsAG card (hepatitis), dengue NS1 card; dengue IgG/IgM; VDRL card (syphilis); glucometer; urine pregnancy, HIV rapid or Coombs; urine sugar (Benedict’s); AFB sputum for TB (in selected clinics)	Lab technician, done in in-house lab, if no lab facilities then referred to public sector taluk or district hospital	For morning samples results by afternoon, doctor seen same day unless test kits/reagents/doctor unavailable, or tests out-sourced	Setting with shorter TATs, but exclusive lab-based testing in context of manpower and equipment shortages leads to delays
	Malaria antigen	Referred to public sector taluk or district hospital or private lab	Patient’s initiative required to get tested, collect results and return to PHC for treatment	
Clinic (private sector)	Glucometer, urine pregnancy	Private provider	Immediate results	Arrangements with private labs nearby ensure POC continuum; other rapid tests not trusted/too costly
Peripheral lab (private sector)	Rapid tests: Widal (typhoid), malaria rapid test (rare), HBsAG, dengue NS1, VDRL (syphilis), glucometer, urine pregnancy dipstick, HIV rapid, HB card, urine sugar (Benedict’s). Others: malaria smears, Mantoux, renal and lipid function, x-rays, scans, urine routine, blood grouping, CBC, blood pressure	Lab technician; done in in-house lab or nearby peripheral lab.	Same day results	Arrangements with private providers nearby ensure POC continuum using older, slower but cheaper methods
	Hormone tests	Outsourced to larger chains of private labs in Bangalore/Mumbai	2 days for tests, results given on 3rd day.	
Hospital (in wards)	Blood sugar with glucometer; urine sugar with dipstick or Benedict’s solution; haemoglobin by blotting paper method, BTCT (Bleeding Time Clotting Time), HIV Tridot, ECG	Staff nurse at in-patient bedside in ward, intensive care unit, emergency or labour ward	Treatment begins at bedside once doctor has seen results	POC continuum ensured for limited tests done in wards
Hospital (in labs)	Rapid tests for HIV, malaria, dengue, HBsAG, VDRL (syphilis), Widal, urine pregnancy, chest x-ray, renal and liver function tests, complete blood count (CBC), ESR (for TB).	Lab technician in in-house laboratory for outpatient department patients	Out-patients collect results from labs, see doctor in afternoon/evening if available; admitted patients: attenders carry samples to labs, nurses collect results if workload permits	Majority of testing is lab-based, high volumes, manpower shortage and different testing/consultation location across hospital campus compound delays
	For TB: AFB sputum, ELISA (tertiary private only)	Lab technician in TB program lab outpatient department block; ELISA outsourced for private secondary care	Patients collect results from separate labs for TB/HIV testing, return results to doctor in afternoon if available	

Legend Table 2:

*AfB* acid fast bacilli, *ANM* auxiliary nurse midwife, *ASHA* accredited social health activist, *BTCT* bleeding time clotting time, *CBC* complete blood count, *CHW* community health worker, *ECG* electrocardiogram, *ELISA* enzyme-linked immunosorbent assay, *ESR* erythrocyte sedimentation rate, *HBsAG* hepatitis B surface antigen, *IgG/IgM* immunoglobulin G/M, *NS1* non-structural protein 1, *PHC* primary health centre, *VDRL* venereal disease research laboratory test

### Home setting

While some tests might be widely available for the public to purchase, hardly any testing occurs in the home.

In our study, the glucometer is the most commonly used diagnostic instrument at home, yet most of the diabetic patients we interviewed did not use it. While it is easily available, only the more affluent can afford the device (Rs1500 or USD24 and upwards) and its testing strips (Rs1200 or USD20 for 50 strips). Even though the results are instantaneous, some laboratory technicians and doctors said that at home, patients do not check their sugar levels daily or at different times of the day, leading to inaccurate or inconclusive results (LT13, 14; PP1, 3, 5, see Table 1 for explanation of interview codes). A private laboratory technician in the rural area described diagnostic practices of his diabetic patients:

*Some of the diabetic patients come by themselves for follow-up and blood glucose level. They come once in 15 or 20 days regularly to monitor. Some patients do self-monitoring with glucometer but it is not accurate – he does not know how to use it properly. (LT 3)*

In our group discussions, diabetic patients could not recall normal or abnormal blood sugar ranges (some of them had never been explained the difference) (FGD#10 DM patients). Instead, most patients with blood sugar conditions rely on monthly visits to doctors or laboratories for routine monitoring or when symptoms such as frequent urination, fatigue, hunger and itchy palms occur (LT1-14). Similarly, urine pregnancy test kits are easily available and more affordable than glucometers, but many patients are unsure of how to use them, bringing the kits to the doctor to administer the test (PP3).

Owning the POC device is not the same as knowing how to use it and having the confidence to interpret the results. Research on medical encounters in India has shown that the doctor-patient relationship is often characterized by medical paternalism, wherein patients’ involvement is limited and dependent on the doctor’s expertise [19, 20]. For testing in the home setting the implications are that patients who are able to afford the diagnostic devices are not or do not feel empowered to use them.

### Community setting

Community Health Workers (CHWs) in India act as a link between the community and public health facilities and are trained in identification of basic key symptoms, awareness creation and referral to health centres. Among CHWs, auxiliary nurse midwifes (ANMs) also conduct deliveries. However, we found that only a very limited number of tests are being used in the field by CHWs. What is more, their use is constrained by the lack of continuous supply of equipment (test kits) due to shortage of funds. Furthermore, once tested, the onus is on the patient to follow-up with treatment at the clinic, while CHWs struggle to convince and support patients, challenged by transportation and manpower constraints.

CHWs mainly do symptom screening, collect malaria blood smears for anyone with fever and sputum samples for tuberculosis microscopy testing at homes or at health camps. In urban areas, ANMs conduct pregnancy tests and haemoglobin with Sahli’s haemoglobinometer as part of ante-natal care check-ups. In rural areas ANMs also do urine albumin and sugar, and malaria rapid diagnostic tests in endemic areas (our study site was a non-endemic area). In addition, HIV mobile testing vans, manned with a laboratory technician and counsellors, conduct HIV rapid tests and Coomb’s test in communities (PO4).

CHWs record patient details in the field and refer those with positive or abnormal readings to the PHC for further testing and treatment. It is the patient’s responsibility to seek further care. Once at the PHC, patients are often faced with non-availability of doctors, long waiting times, repeat tests to confirm field tests, and informal payments for investigations.

The TATs for samples that are being tested at public clinics depend on the clinic setting (see below). Where transportation is lacking, samples may dry up before they reach the clinic and have to be re-taken. Several CHWs reported stock-outs and non-availability of test kits (FGD#3ASHA) and fear for their personal safety when dealing with social problems like alcoholism and domestic violence.

*In the last 4 years, we have been given the [urine pregnancy test] kits twice. After that they have not given it to us and we have been calling the patients here [to the PHC]. (FGD #3 ASHAs, Respondent 0)*

The results of sputum samples and blood smears for malaria are communicated to the CHW over the phone, yet may not always reach the patient given the large population CHWs have to cover and the limited manpower and transportation resources they have.

CHWs told us that tests conducted at the doorstep along with regular home visits can be a convincing tool to seek care at PHCs (as results are immediate, visible and free).

*It is hard to bring patients to the PHC. The solution is that we should be able to do it in the village itself near their home or at the doorstep, whether it is treatment or testing. For small things at least that should be done and major problems that we can bring them to PHC. (FGD #3 ASHAs, Respondent 1)*

Yet, the CHWs in our study also warned that major challenges would remain despite testing at the doorstep, such as counselling patients on the spot to follow-up with tests and treatment, especially for stigmatized diseases like HIV and TB (CHW1&2), the cost for patients in accessing care (transport, food and losing a day’s wages) and lack of facilities or manpower at the PHCs (CHW1&2, FGD#7LW, FGD#2ANM).

Community health workers (CHWs) are often seen as important users of POC tests [21]. Our results confirm that a doorstep delivery approach by CHWs can lessen the onus on patients in accessing care, as has been shown in general health service delivery in Bangladesh, [22]. Yet, our results also show that health system challenges, such as lack of equipment and infrastructure support [23], undermine the potential of CHWs to link between patients, communities and healthcare providers. Even if available, easy to operate and rapid, the diagnostic technology alone cannot overcome these problems.

### Public clinic setting

We found that a lack of funds restricts the availability of rapid test kits in public clinics. Where rapid tests are available, the test and treat cycle is not completed in one patient encounter: rapid tests are conducted in small laboratories challenged by workload and manpower, resulting in too long TATs, and patients have to be referred elsewhere for confirmatory testing for HIV and TB, all of which can cause patient attrition.

The public clinic setting is represented by the most basic unit of the Indian public health system, the PHC in rural areas (urban health centres in urban areas) staffed with one qualified physician and/or Ayush medical officer, one to three staff nurses, two laboratory technicians, four CHWs and one pharmacist. They provide preventative and curative treatment and implement national disease control programs. All PHCs usually have attached laboratories for conducting basic tests. Every third or so PHC is equipped with TB microscopy, but they do not have scanning or X-ray facilities. In reality, PHCs are often understaffed with insufficient laboratory facilities and funds for testing kits and laboratory consumables. This means that patients or their samples are often sent to the next level of care ((sub-)district hospital).

Most tests at the clinic setting are conducted in the PHC laboratory by a trained technician, including malaria smears, HbsAg card test (hepatitis), HIV rapid and Coomb’s tests, dengue NS1 card test and dengue through IgG and IgM lab tests, urine dipstick, urine sugar testing with Benedict’s solution, urine albumin, and total and differential count of white blood cells. In some PHCs, the HIV test is done by a technician sent by the HIV/AIDS program on ante-natal care day (LT2, LT5). After 5 pm, when laboratories close, staff nurses perform some tests, such as glucometer, malaria smears and HIV rapid tests for women in labour without ante-natal care or HIV reports (SN1). According to doctors, the urine pregnancy test is the only rapid test done during consultation, but confirmatory tests are sent to the laboratory (MO1&2).

High workloads lead to delays and backlogs. Doctors see about 30 follow-up ante-natal care cases and 50 to 90 new patients per day (MO1,2). They send about half of all patients seen for laboratory tests (LT20&19). Laboratory technicians investigate 800 to 900 smears for malaria a month including those sent by CHWs (LT,5, 19, 20), and about 200 routine random blood sugar tests (with a glucometer) per month. One laboratory technician (LT2) said she does 35 ante-natal care test packages a day (each including HBsAG via card, blood sugar with glucometer, VDRL card test (syphilis) and blood grouping, with a total TAT of one hour).

Depending on patient load and doctor availability, TATs can get delayed if doctors cannot be seen on the same day.

*For HIV, if the PHC has not procured the Tridot card test for HIV, they use the Coombs Aid test which takes more than an hour for a result. So patients coming in the morning are told to return by 2 to 3 pm and those who give samples in the afternoon are told to come the next day which could cause patient loss* (LT19).

In the interim, some form of empirical treatment could have begun, but no conclusive diagnoses would have been made.

Patients who have tested positive for HIV at the clinic have to go to a hospital for confirmatory testing (using ELISA or rapid card tests) often without knowing their result due to absence of trained counsellors at clinics (LT18, 19).

*If HIV is positive (…) we will not tell the patient…there is no counseling here and also we do not have the two confirmatory tests here, so we send them to the ICTC center or to the general hospital* (LT19)*.*

The referral system is weak and informal, at which point patient loss to follow-up occurs. A systematic review of studies on patient loss between testing, confirmatory testing and treatment for HIV/AIDS in Sub-Saharan Africa found that loss to follow-up happened for more than two thirds of patients tested positive. Lacking information system between facilities was found to be among the factors causing patient loss, followed by patients being asymptomatic, cost involved to access testing and fear of social stigma [24].

Imbalanced resource allocation, limited physical access to quality health services and inadequate human resources [23] as well as inability to translate funds into public health services [25] are well documented challenges of India’s primary health clinics. Our results show that delays in TATs caused by equipment, workload and manpower challenges in public laboratories are even more disastrous in settings with weak counselling and referral systems.

### Private clinic setting

Coordination between private doctors and private laboratories encourage the use of the latter to perform tests, and the alignment of consulting times. Private practitioners tend to order chemical analysis tests over rapid ones because of high costs and lacking faith in the accuracy of the latter.

Private clinics in this study range from small outpatient clinics with basic facilities, to clinics that manage seven in-patients with small in-house laboratories, to larger nursing homes with wards, in-house laboratories and operating theatres. Accordingly, the qualifications of these providers range from basic in Ayurvedic or allopathic medicine in small clinics to specialisation degrees in larger centres. The unqualified providers often represent the first point of contact for patients in remote areas.

Most private clinics are open for out-patients in the morning and again in the evening. Providers see an average of 60 patients a day (around 100 in larger nursing homes) and spend between 5 to 15 minutes with each one. Average consultation charges range from Rs100 to Rs150 (equivalent to USD1.6-2.5), with Rs30 (USD0.5) for unqualified rural providers, up to Rs300 (USD4.5) in larger nursing homes, although some fees are waived for the poorest. Patients from far off villages also seek care in private clinics when facilities closer to home fail them.

Private doctors rarely do any testing in their own clinics and order the majority of tests in nearby laboratories. According to private laboratory technicians (LT1-6), the majority of doctors in private clinics get ‘kick-backs’ from laboratories for ordering tests, a commission ranging from 20 to 40 % of the price paid by patients (PP1,6,11). Most doctors preferred not to admit to having received a commission and instead claimed not to keep track of how many tests they order. When they admitted it, they claimed to put the money back into the clinic’s infrastructure (e.g. to buy an ambulance), fund a local charity, or allocate it for the poorest patients (PP 2–3,6,7,10). As a doctor in a small rural clinic explained:

*We will not worry about how much is coming [as commission]. Whatever they [lab*oratorie*s] give, we will keep it for the poorest patients or donate to a mosque or madrasa.(PP6)*

We found that private clinics and laboratories often coordinate their opening hours, allowing patients to be seen by the doctor in the morning, get tested at the laboratory nearby during the day and return to the clinic in the afternoon or evening with the results. This ensures a POC continuum within the same day.

Pregnancy tests and glucometer (and ECG machines for larger clinics) are the only tests commonly used in private clinics on the spot with a TAT of minutes. These tests are easily available at pharmacies (chemists) and some patients bring them to the doctor (see home setting). This shows not only the commonality of these tests (and their associated conditions), but again that some patients feel the need for a doctor’s expertise to conduct tests or read their results.

Tests usually ordered by private doctors in local laboratories are: Widal (rapid slide test for typhoid), haemoglobin by Sahli’s method (or semi-/automated in bigger laboratories), CBC (complete blood count), platelet count, HbsAG (card test) for fever with a TAT of 30 minutes to 1 hour for results while card tests produce instant results; Mantoux, chest X-ray and microscopy for TB (done in public sector laboratories) with a TAT of 3 days for final confirmation; renal and liver function tests with TATs of 2 days, blood sugar, albumin, bilirubin, bile salts done using chemical analysis in the laboratory with TATs of 30 minutes (in-house or referred outside depending on facilities present); tests like thyroid hormone levels take 3–4 days and are sent to large private laboratories.

Many private providers lack faith in the accuracy of rapid test kits that are often too expensive for patients (for instance Rs800 for a dengue NSI card test). Instead, they order laboratory tests that use chemical analysis (i.e. to confirm pregnancy and blood sugar) and microscopy (PP1, 13). Unqualified practitioners in rural areas we spoke to generally administer steroid injections for temporary relief, but also intravenous fluids and insulin injections for previously diagnosed diabetic patients (patients bring their own syringes). On the spot, they only check blood pressure and temperature and send cases of fever and diarrhoea to laboratories nearby (PP8&9).

With poorer patients, many private practitioners in small clinics prefer clinical diagnosis over investigations that patients cannot afford.

*The reason I do not prefer to go for investigations is the high cost. Most of the patients cannot afford it and we don’t like to force them. (PP5)*

Most providers claimed that they refer TB suspects (prolonged cough, positive X-rays) to the government TB facility for confirmation and free treatment. Our interviews with TB patients showed that this only happened after repeated misdiagnosis and mistreatment with frequent provider change (e.g.. TB Patient 5). HIV is diagnosed using rapid tests in laboratories but for confirmatory ELISA tests, patients are sent to the government’s national HIV/AIDS facility. Those referrals are often done without revealing positive results to patients to avoid losing them (LT6,18).

*If they are illiterate we don’t tell them we are sending for HIV or TB – they will get scared and won’t come back with the result or get the test done. (PP12)*

Patients are referred to larger hospitals also for more complex cases such as pregnant women with pedal edema (PP3). Since there is no formal referral system between private and public providers and often limited counselling, loss to follow-up can occur with similar consequences for the POC continuum as discussed for public clinics.

In light of a poorly funded public sector, India’s large and unregulated private healthcare sector is the preferred choice by poor and better-off Indians alike, although many private providers perform unnecessary diagnostic tests and surgical procedures with at times questionable quality [26–29]. Our interviews with TB patients, for instance, show that delays caused by repeated misdiagnosis are particularly long if private providers are visited first [30–32]. The limited use of tests on the spot by private doctors and their doubts about the accuracy of rapid tests and cost for patients confirm earlier studies on clinicians’ attitudes towards POC testing [5, 33]. While kick-backs between private doctors can encourage inadequate tests to be widely used [29] and foster mismanagement of TB [31], we find that the POC continuum is often ensured because of these arrangements.

### Peripheral laboratories (private)

The high cost of rapid kits and reagents prohibits their use in poorer neighborhoods where most small laboratories are located. But due to smaller volumes, these laboratories are able to maintain a one hour TAT using older methods. Larger laboratories do use rapid tests, especially for self-referrals, but patients still have to collect results and return to a doctor. Coordination between doctors and laboratories helps maintain the POC continuums, as do arrangements between polyclinics and in-house diagnostic centres.

Our study included a variety of private laboratories from small one-roomed ‘shops’ performing a simple array of tests, to medium-sized facilities, to large chains of diagnostic centres with headquarters in the state’s capital. The smaller laboratories have basic infrastructure, some with only benches and no waiting areas or beds. All seemed to have a refrigerator and a power back-up system, though only for a few hours. Medium-sized laboratories have waiting areas, but only small one-roomed testing facilities where equipment is stored. The larger more well equipped laboratories have at least one chemical analyzer (semi-automatic or automatic), a centrifuge, incubator, microscope, and two to three technicians.

Smaller laboratories have a workload of around 10 to 30 patients a day, with medium-sized facilities working with around 50 samples a day. Most laboratories are open from 8 am until 10 pm. The morning rush of regular diabetic patients testing fasting blood sugars tends to delay TATs from one hour to two. As the day progresses and patients are being referred from the clinics, the laboratories perform a variety of tests including: HB card method, random blood sugar (glucometer or manual), urine dipstick or chemical analyser, platelets, CBC, malaria using microscope (rarely rapid card test), blood grouping, HBsAG, VDRL, typhoid slide (Widal), TB Mantoux and HIV Tridot. The TATs for most tests is one hour. The TAT of a rapid test extends to one hour if ordered with a battery of other tests using older methods.

In all laboratories, patients can collect results in the afternoon for samples given in the morning and samples given in the afternoon are collected in the evening or the next day. Patients collect reports directly from the laboratories and then queue at their doctor for interpretation of results. Some patients are too poor to pay for results and fail to collect them. To counter their financial losses, laboratories tend to ask patients for partial payment upfront.

The high costs of rapid card tests compared to older methods (Table 3) is a key reason why smaller laboratories in poor neighborhoods do not use rapid tests. An exception is the newer slide test for typhoid which takes 20 minutes and is cheap (Rs50, USD0.85) and thus preferred over the older tube test which takes 24 hours and is more expensive (Rs250, USD 4.15). Some expensive tests like cholesterol and lipid profiles are rarely done except for those with private health insurance. The costs of testing equipment (analyzers, microscopes), rapid test kits and reagents force smaller laboratories to invest in older, cheaper, and potentially less accurate methods. At the same time, laboratories face stiff competition from polyclinics that have started in-house diagnostic centres.

**Table 3:** Cost of tests in rural and urban, private peripheral labs (LT #4-9, 12,17, PP#2)

Name of test	Cost
Glucometer	Rs40 (USD0.65)
Blood grouping	Rs45 (USD75)
Urine test	Rs50 (USD0.85)
Typhoid slide (Widal)	Rs50 (USD0.85)
Haemoglobin	Rs50 (USD0.85)
FBS, PPBS blood sugar (manual)	Rs60 (Rs25) (USD1, USD0.40)
Platelet	Rs80-Rs150 (USD1.35-USD2.50)
Albumin	Rs100 (USD1.65)
Urine pregnancy test	Rs100 (USD1.65)
Malaria smear	Rs100 (USD1.65)
Smear for acid fast bacilli (TB)	Rs100 (USD1.65)
Mantoux	Rs100 (USD1.65)
VDRL (syphilis)	Rs100 (USD1.65)
Sputum	Rs120 (USD2)
HbsAG card (hepatitis)	Rs120 (USD2)
Coombs test (HIV)	Rs150 (USD2.50)
HIV rapid (Tridot)	Rs200 (USD3.30)
Complete blood count (CBC)	Rs250 (USD4.15)
Typhoid tube	Rs250 (USD4.15)
Malaria card	Rs300 (USD5)
Blood/Urine/pus Culture & sensitivity	Rs250 to Rs350 (USD4.15-USD5.85)
HIV 1 & 2 (serology)	RS350 (USD5.85)
Thyroid hormone assays	Rs350 (USD5.85)
Fever profile	Rs400 (USD6.65)
Lipid profile	Rs400 (USD6.65)
Renal profile	Rs600 (USD10)
TB	Rs650 (USD10.85) (X-ray: Rs200 (USD3.35); CBC: Rs250 (USD4.15); Sputum: Rs100 (USD1.65); Mantoux: Rs100 (USD1.65))
Dengue NS1 card test	Rs750 (USD12.50)
Ante-natal profile (haemoglobin, random blood sugar, HBsAG, VDRL, HIV, urine routine, blood grouping)	Rs750 (USD12.50)
TB serology	Rs900 (USD15)
Polymerase chain reaction (PCR)	Rs1500 (USD25)
Fertility related hormone assays	Rs15,000 (USD249.75)

The diagnostic process between clinics and laboratories is at times altered on patients’ or laboratory technicians’ initiatives: patients with fever access laboratories specifically requesting malaria, typhoid and dengue tests to avoid queuing at the doctor’s clinic first (LT 6,16). A laboratory technician illustrates: “*The fever comes they think it is typhoid or dengue so they ask us, we send them saying consult a doctor and come. (LT18)* While public sector laboratory technicians have been instructed to test anyone with fever, private laboratory technicians advise the patient to see the doctor first. But given that most laboratories are under pressure to make a profit, they are likely to do the patient’s bidding and also interpret test results so that patients can choose if they want to consult the doctor.

*‘I will do three tests but I will not be responsible for what the doctor says’. When they come without consulting the doctor, we will have to tell them that. ‘For fever, there are many tests, we cannot do all of them, we can do two or three of the tests- but if you force us’. (LT18)*

Yet, many laboratory technicians refuse self-referred patients for HIV testing without a doctor’s referral as communicating a positive result causes more problems for the laboratory without proper training or counselling facilities (e.g. to newly married couples in case one partner tested positive or when patients are unwilling to accept a result and might blame the laboratory for it) (LT18, 19).

*Sometimes people come on their own for HIV and we just reject the test and (…) send them away saying ‘We cannot do it, it is done free at the government hospital.’ … They can go there and get it done, we tell them, ‘We do not want your money, we do not want you to get in to problem and then create problems for us’ (LT18)*

In private laboratories, counselling for HIV does not take place and patients are rarely told they are being sent to the public HIV/AIDS program for confirmation of HIV because of social stigma; when the rapid results are positive for HIV, most laboratories tell the patient that the test wasn’t available or the machine was not working.

It is estimated that India has over 100’000 diagnostic laboratories with only 1 % being accredited [34]. Despite the lack of quality control and accreditation, India’s large numbers of small peripheral laboratories are frequently accessed by patients and thus important players in POC testing. We show how self-referrals and the time-saving tactics used by patients turn the diagnostic process on its head: completing tests before visiting a doctor. Furthermore, our results highlight that these laboratories manage to ensure POC continuums by using older methods than rapid tests and ensuring coordination with the treating provider. The tendency for keeping patients uninformed and maintaining medical paternalism in doctor-patient relationships [19, 20] is extended to patient-laboratory technician encounters, adding to confusion and patient attrition

### Hospital setting

Tertiary hospitals (public, private, medical colleges) tend to use more rapid tests than most other settings, in the wards (for in-patients) and in the laboratory. Yet, the majority of rapid tests are done in laboratories. This fails to use them to their full potential and jeopardizes the POC continuum with outpatient department (OPD) and in-patients having to wait half a day for results (3–4 hours) unless it’s an emergency, in which case the TATs are immediate. Particularly for HIV and TB tests, patients have to shunt between locations risking further delays and loss to follow-up.

Poor people tend to seek care in public hospitals [35] as treatment is free, although quality of services may be poor and there are additional costs such as travel and food. Only the middle and upper classes can afford treatment at private hospitals, while private medical colleges operate at the mid-price range, offering subsidized rates for all people. The latter tends to offer better quality of care than the public sector at a cheaper price than private hospitals.

Most doctors in hospitals, especially specialists, say they rely less on diagnostic tests and more on clinical findings. There is no defined protocol for ordering tests, other than less obvious conditions may require a battery of tests while the more clinically evident conditions require less tests (SP 2,9,10,14). However, private hospitals catering to a higher socio-economic clientele tend to order a series of tests, while those catering to poorer patients order the basic minimum tests to save the patient money.

*I hate the word ‘Corporate’ because corporate hospitals charge a lot and I do not want to follow that…Here we try to think what less investigations, less tests, less medications are required…to arrive at a diagnosis to treat the patient at an affordable cost.(SP 9)*

In public and private hospitals, diagnostic tests include: malaria smear and rapid test (only in an emergency due to high costs), dengue NS1 and ELISA, urine routine, Widal, renal and liver function tests, complete blood count, platelet count and Tridot and ELISA for HIV. All tests are done in the laboratory except RBS by glucometer, ECG, urine dipstick test and HIV Tridot for pregnant women which are done in wards by staff nurses and post-graduate medical students.

All TB patients are offered free HIV testing in public hospitals. HIV and TB tests are processed in separate laboratories: patients need to go to those rooms in the OPD block and return to collect reports. Patients being tested for HIV, TB as well as co-morbidities have to visit up to three different locations for tests and to collect results, rather than a single visit at a centralised laboratory. While private secondary and tertiary hospitals may have centralised laboratories, some private hospitals send patients to public hospitals for confirmatory testing for HIV (ELISA) and TB (AFB sputum) and/or treatment initiation (Lab Manager 1, PP5, TB patient 4).

On average, in all hospital settings (rural and urban, and public and private), the TAT for diagnostic tests done in the in-house laboratory is about three to four hours between the morning (9 am to 2 pm) and afternoon OPD (2 pm to 4:30 pm). Patients or their attenders have to collect laboratory results and then see the doctor in the afternoon OPD for further decisions to be taken. The TAT for specialised tests (culture and hormonal tests) takes 24 hours in private tertiary hospitals (due to electronically available results (Lab manager 1)) and from 48 to 72 hours in public tertiary and private secondary hospitals.

Delays in TAT can happen when repair of equipment and purchase of reagents are delayed; also rapid tests are too expensive for public hospitals, and a high turnover of laboratory technicians in all types of hospitals costs time and money (SP9). Delays can also happen if patients cannot find doctors in the afternoon hours or if they need to get tested across different laboratory facilities on campus. Some patients come to hospitals after unsuccessfully seeking care elsewhere and may not give accurate accounts of their history forcing doctors to repeat tests.

Further delays can happen due to a lack of manpower to transport samples and reports to and from laboratories, and misplacement of records. To overcome that challenge, private reference laboratories send ‘collection boys’ daily to collect samples at private secondary care hospitals and bring back results after 48 hours (PP5, LT 9, SP 9). In public tertiary care hospitals, samples are referred to outside reference laboratories only if patients can afford it and taken there by the patient’s relatives (SP 1, 2).

Moreover, where some doctors also practice in private clinics, patients may have to follow doctors to a different location or end up in the wrong one, another disruption to the single patient encounter (SP 4,5,7, 11,16, Hospital Manager 1).

Our finding that the majority of rapid tests are done in hospital laboratories and thus are not used to their full potential, highlights that rapid tests are not always completed rapidly [36–38]. Manpower, workload, equipment, facilities and cost can cause additional delays further extending the TATs of (rapid) tests conducted in hospital laboratories and compromising their potential to ensure the POC continuum. The specific testing infrastructure of vertical disease programs can further compound diagnostic delays. Integrating vertical disease control strategies into the general health systems [7] would therefore need to involve basic infrastructural adjustments, such as centralizing laboratories across hospital campuses.

## Conclusions

In this study, we have for the first time used qualitative methods to document the use of POC tests across the spectrum of settings and diseases in the Indian health care system.

Successful POC testing hardly occurs in any of the five settings. Our findings suggest that rapid tests are not being used to their full potential as POC testing programs: instead, they are used in laboratories of public clinics and hospitals with too long TATs, leading to patients having to come back the next day. In the public sector, lab-based testing in a context of manpower and equipment shortages leads to delays and disruptions in the POC continuum. In settings where TAT would be shorter (e.g. in communities, in small peripheral laboratories), rapid tests are not being used because of non-availability (public) and too high cost of reagents (small private laboratories), or lack of faith in their accuracy (private clinics). In settings where POC continuums are being ensured, the tests used are not always rapid tests. Private laboratories and clinics ensure a POC continuum without specific rapid technologies by relying on older and often cheaper methods and aligning opening hours and their localities through established coordination arrangements to keep TATs at a minimum.

In homes, cost of testing devices and strips restrict testing to the affluent and educated. While ideally the use of POC testing at home should ensure that patients only visit a laboratory or a doctor with positive or abnormal results, we found that patients who use these tests still visit doctors for confirmation or to read results correctly. This is compounded by widespread medical paternalism and lack of counselling in both doctor - patient and laboratory technician – patient encounters. Thus, POC tests are not fulfilling their purpose in this setting and future home-testing will require robust support and counselling strategies that are sufficiently linked to patients’ homes [39].

In all settings, the TATs of all tests are potentially compromised because the onus is on patients to ensure POC continuums: patients often have to travel to peripheral laboratories (or a different facility in the same setting) to give samples and bring results back to doctors. POC continuums are further compromised by weak referral systems between providers, lack of functioning support for patients (CHWs constrained by workload and transportation) and patient loss to other providers.

We conclude that the mere availability of rapid so-called POC tests does not guarantee their scale up or use in a way that preserves the integrity of the single patient encounter to facilitate immediate treatment. In reality, it is compromised by intervening factors such as costs, availability, arrangements that encourage use of laboratories, disbelief in accuracy of rapid tests, reliance on patient initiative to collect results and follow-up with treatment and patients switching providers. Patients’ self-referrals and time-saving tactics as well as laboratory technicians’ additional testing or counselling can turn the diagnostic process on its head with unclear implications for the POC continuum.

Our study confirms what has been argued elsewhere, that tests by themselves cannot be called POC or not; it is how the tests are deployed or implemented that define POC testing [1]. Integration within the existing health system is crucial. Our findings show how arrangements between providers matter for the POC continuum. Similarly, systems for rapid reporting of test results to care providers, real-world workflow patterns, infrastructural set-ups linked to disease control strategies and economic/incentive structures are as important as the test itself. Designers of rapid tests, global health experts and policy-makers need to account for these ground realities and modify design strategies and policies accordingly.

In this exploratory approach we have identified how tests are used across the five different settings. Future studies could examine these different uses on a larger scale or for specific diseases and tests.

## Additional file

Additional file 1:
**Interview guide POC testing in India.** (DOC 75 kb)

## References

[1] Pant Pai N, Vadnais C, Denkinger C, Engel N, Pai M (2012). Point-of-Care Testing for Infectious Diseases: Diversity, Complexity, and Barriers in Low- And Middle-Income Countries. PLoS Med.

[2] Zar HJ, Workman L, Isaacs W, Munro J, Black F, Eley B (2012). Rapid Molecular Diagnosis of Pulmonary Tuberculosis in Children Using Nasopharyngeal Specimens. Clin Infect Dis.

[3] Negin J, Wariero J, Mutuo P, Jan S, Pronyk P (2009). Feasibility, acceptability and cost of home-based HIV testing in rural Kenya. Trop Med Int Health.

[4] Prost A, Griffiths CJ, Anderson J, Wight D, Hart GJ (2009). Feasibility and acceptability of offering rapid HIV tests to patients registering with primary care in London (UK): a pilot study. Sex Transm Infect.

[5] Jones CH, Howick J, Roberts NW, Price CP, Heneghan C, Pluddemann A (2013). Primary care clinicians’ attitudes towards point-of-care blood testing: a systematic review of qualitative studies. BMC Fam Pract.

[6] World Health Oganization (2007). Everybody’s business: Strengthening health systems to improve health outcomes: WHO’s framework for action.

[7] Collins CD, Green AT, Newell JN (2002). The relationship between disease control strategies and health system development: the case of TB. Health Policy.

[8] Boehme CC, Nicol MP, Nabeta P, Michael JS, Gotuzzo E, Tahirli R (2011). Feasibility, diagnostic accuracy, and effectiveness of decentralised use of the Xpert MTB/RIF test for diagnosis of tuberculosis and multidrug resistance: a multicentre implementation study. Lancet.

[9] Tucker JD, Yang LG, Zhu ZJ, Yang B, Yin YP, Cohen MS (2010). Integrated syphilis/HIV screening in China: a qualitative analysis. BMC Health Serv Res.

[10] Mabey DC, Sollis KA, Kelly HA, Benzaken AS, Bitarakwate E, Changalucha J (2012). Point-of-Care Tests to Strengthen Health Systems and Save Newborn Lives: The Case of Syphilis. PLoS Med.

[11] Palamountain KM, Baker J, Cowan EP, Essajee S, Mazzola LT, Metzler M (2012). Perspectives on introduction and implementation of new point-of-care diagnostic tests. J Infect Dis.

[12] Zumla A, Cobelens F (2012). Operational research and MDG tuberculosis control targets. Lancet Infect Dis.

[13] Mann G, Squire SB, Bissell K, Eliseev P, Du Toit E, Hesseling A (2010). Beyond accuracy: creating a comprehensive evidence base for TB diagnostic tools [State of the art]. Int J Tuberc Lung Dis.

[14] Engel N, Pai M (2013). Tuberculosis diagnostics: Why we need more qualitative research. Journal of Epidemiology and Global Health.

[15] Braun V, Clarke V (2006). Using thematic analysis in psychology. Qual Res Psychol.

[16] Eisenhardt KM, Graebner ME (2007). Theory building from cases: Opportunities and challenges. Acad Manage J.

[17] Rubin HJ, Rubin IS (2005). Qualitative Interviewing: The Art of Hearing Data.

[18] Engel N, Ganesh G, Patil M, Yellappa V, Pant Pai N, Vadnais C (2015). Barriers to point-of-care testing in India: Results from qualitative research across different settings, users and major diseases. PLoS ONE.

[19] Fochsen G, Deshpande K, Ringsberg KC, Thorson A (2009). Conflicting accountabilities: Doctor’s dilemma in TB control in rural India. Health Policy.

[20] Datye V, Kielmann K, Sheikh K, Deshmukh D, Deshpande S, Porter J (2006). Private practitioners’ communications with patients around HIV testing in Pune, India. Health Policy Plan.

[21] Hawkes M, Katsuva J, Masumbuko C (2009). Use and limitations of malaria rapid diagnostic testing by community health workers in war-torn Democratic Republic of Congo. Malar J.

[22] El Arifeen S, Christou A, Reichenbach L, Osman FA, Azad K, Islam KS (2013). Community-based approaches and partnerships: innovations in health-service delivery in Bangladesh. Lancet.

[23] Balarajan Y, Selvaraj S, Subramanian SV (2011). Health care and equity in India. Lancet.

[24] Rosen S, Fox MP (2011). Retention in HIV Care between Testing and Treatment in Sub-Saharan Africa: A Systematic Review. PLoS Med.

[25] George B (2011). India’s Public Health: A Financial Aetiology. Econ Pol Wkly.

[26] Sengupta A, Nundy S (2005). The private health sector in India. BMJ.

[27] De Costa A, Johansson E, Diwan VK (2008). Barriers of Mistrust: Public and Private Health Sectors’ Perceptions of Each Other in Madhya Pradesh, India. Qual Health Res.

[28] Peters DH, Muraleedharan VR (2008). Regulating India’s health services: To what end? What future?. Soc Sci Med.

[29] Jaroslawski S, Pai M (2012). Why are inaccurate tuberculosis serological tests widely used in the Indian private healthcare sector? A root-cause analysis. Journal of Epidemiology and Global Health.

[30] Rajeswari R, Chandrasekaran V, Suhadev M, Sivasubramaniam S, Sudha G, Renu G (2002). Factors associated with patient and health system delays in the diagnosis of tuberculosis in South India. Int J Tuberc Lung Dis.

[31] Udwadia ZF, Pinto LM, Uplekar M (2010). Tuberculosis Management by Private Practitioners in Mumbai, India: Has Anything Changed in Two Decades?. PLoS One.

[32] Sreeramareddy CT, Qin ZZ, Satyanarayana S, Subbaraman R, Pai M (2014). Delays in diagnosis and treatment of pulmonary tuberculosis in India: a systematic review. INT J TUBERC LUNG DIS.

[33] Agusti C, Fernandez-Lopez L, Mascort J, Carrillo R, Aguado C, Montoliu A (2013). Acceptability of rapid HIV diagnosis technology among primary healthcare practitioners in Spain. AIDS Care.

[34] Dey S (2014). Lack of regulation haunts India’s test labs. Business Standard.

[35] Prinja S, Kumar MI, Pinto AD, Jan S, Kumar R (2013). Equity in Hospital Services Utilisation in India. Econ Pol Wkly.

[36] Chandler CI, Hall-Clifford R, Asaph T, Pascal M, Clarke S, Mbonye AK (2011). Introducing malaria rapid diagnostic tests at registered drug shops in Uganda: limitations of diagnostic testing in the reality of diagnosis. Soc Sci Med.

[37] Clouse K, Page-Shipp L, Dansey H, Moatlhodi B, Scott L, Bassett J (2012). Implementation of Xpert MTB/RIF for routine point-of-care diagnosis of tuberculosis at the primary care level. S Afr Med J.

[38] Losina E, Bassett IV, Giddy J, Chetty S, Regan S, Walensky RP (2010). The “ART” of Linkage: Pre-Treatment Loss to Care after HIV Diagnosis at Two PEPFAR Sites in Durban, South Africa. PLoS One.

[39] Pant Pai N, Klein MB (2008). Are we Ready for Home-based, Self-testing for HIV?. Futur HIV Ther.

