# Supplementary material for: Point-of-care testing in India: missed opportunities to realize the true potential of point-of-care testing programs
Source: BMC Health Serv Res. 2015 Dec 14;15:550. doi: 10.1186/s12913-015-1223-3 (PMC4677441; doi:10.1186/s12913-015-1223-3)
Supplement: Additional file 1: — Interview guide POC testing in India. (DOC 75 kb) [file 12913_2015_1223_MOESM1_ESM.doc]

**Interview guide POC testing in India**

The aim of the key-informant interviews is to gain insights into the following aspects:

- Existing diagnostic practices at the point of care
- Barriers to widespread use of POC tests within the diagnostic ecosystem
- Suggestions to overcome barriers identified
- Different understandings and ideals of a POC test

*Background*

| - Questions   Probes | Information you should receive/collect |
| --- | --- |
| - What do you do in your daily work? | Background, function, daily work of the key informant |
| - What kind of setting is this? | Type of practice (if applicable), number and kind of other support staff, infrastructure |
| - What are the main diseases you see? - Number of tests you conduct? | Prevalent infectious diseases, number of patients/samples per day |
| - For how many patients do you order tests ? | Ratio test order and treated clinically |

*Existing diagnostic practices at POC and potential barriers to POCT (probe for examples and reasons)*

| - How do you diagnose? | Steps performed  Understanding of diagnosis |
| --- | --- |
| - What diagnostic tests do you use/order?   How? Why? When? How much? Examples? How many? Time required? Who is involved? Use of rapid test technology? Challenges? | Processes, steps involved  particularly: HIV, malaria, syphilis, hepatitis, and dengue, TB |
| - How do you interact with the laboratory/referring provider? | Process, referral, interaction, challenges |
| - What happens when result is available? | Process. who acts on result, when  Influence of test result on clinical practice (for TB) |
| - When can you ask a test and can make treatment decision on the spot (while the patient waits)? | For which diseases? In which cases?  Examples, Barriers |
| - How are CHWs involved? | their tasks related to testing & treatment, use of tests, potential for diagnostic testing |
| - What are the costs involved?   How? | For instance: for each test, kick-backs, user charges, reimbursement, willingness to pay clinician |
| - How do you assure quality? | Understanding of quality (what is it? what matters?) Mechanisms, processes, challenges |
| - How do you record data? | On patients & test results  Processes, notifications  Actors involved |
| - How are patients informed /counseled? | Understanding, processes, actors involved, challenges in interacting with patients |
| - Have you changed the diagnostic set-up at one point? | Processes  Challenges  Actors involved |
| - What are your main challenges in diagnosing? | Unmet needs  You can also probe for challenges mentioned by other actors/ in FGDs |
| - How do you deal with the challenge? |  |
| - What is a good diagnosis? | Understanding of ideal  Situation, view on diagnosing |

Different understandings and ideals of a POC test

| - Imagine a test that would allow a treatment decision within one clinical encounter   How long would patient wait? |  |
| --- | --- |
| - What would be criteria of an ideal TB test? - What would you suggest to test developers? | understand reasons for answers |
| - What test would you conduct here? |  |
| - What could be diagnosed at different levels? |  |
| - What could be barriers to POC testing of infectious diseases? | Any additional comments  Understanding of POC |
| - THANK YOU - Can you suggest someone else to talk to? |  |

**Patient interview guide**

The aim of the patient interviews is to gain insights into the following aspects:

- treatment seeking behavior: reasons for seeking care, when, where, perceptions of quality, symptom identification and interpretation; interaction with health personnel; social support factors; and financial and structural barriers
- perceived needs of a POC test by patients
- Self-testing HIV & diabetes

| - Questions   Probes | Information you should receive/collect |
| --- | --- |
| - What is your occupation? | Background, age, level of education, economic status |
| - Tell me how you came to know you had disease X   (Tell me everything that happened before you started treatment) | Get the person talking, let the person tell their own story from his/her own perspective |
| - What were your symptoms? - How long did you wait to seek care? Why? | reasons for seeking diagnosis, potential for delays |
| - Where did you go first/last?   Why? Who/what influenced you? | Reasons to seek service provider, understanding of what is a good doctor |
| - What happened there?   Who did you meet? How long did you wait?  Was a test explained?  What sample was taken? What happened next?  When did you see a doctor?  What did doctor do?  Did you get a report?  When?  How was result communicated?  Where did you wait?  When was treatment initiated?  How?  What were the costs? | Minute details of the diagnostic process &treatment initiation  Probe for the detailed steps or ask  What happened next? |
| - Did you visit another provider?   Why? How long did you wait?  How long were you with this provider?  What happened there? (🡪see above)  Repeat this question till no more providers | sequence of providers visited  reasons  duration of medical care and time gap between successive providers |

| - Why did you change providers?   Were you not happy with it? | reasons for discontinuation of medical care/change of providers |
| --- | --- |
| - What is a good doctor?   How do you know? | Understanding of quality |
| - How long are you waiting for doctor, for lab result?   How long are you able to wait/willing to wait? |  |
| - What is a good diagnostic test/process?   What is important to you? | Understanding of ideal test |
| - Why do you think you have this disease? | understanding of TB, cause of TB/illness |
| - Did you tell your family?   How did they react?  How did your community react?  Why? | responses of community, family, environment, perceived stigmatization |
| - Have you ever tested yourself?   How?  Experiences?  Challenges? |  |
| - Would you test yourself with such tests?   Why?/Why not? | User feedback on dummy overall reactions, pro/con, comparison with current device, useability, feasibility |
